# Prevalence and clinical profile of chronic pain and its association with mental disorders

**DOI:** 10.11606/S1518-8787.2017051007025

**Published:** 2017-11-07

**Authors:** Flávia Garcia Pereira, Mariane Henriques França, Maria Cristina Alochio de Paiva, Laura Helena Andrade, Maria Carmen Viana

**Affiliations:** I Universidade Federal do Espírito Santo. Programa de Pós-Graduação em Saúde Coletiva. Vitória, ES, Brasil; II Universidade de São Paulo. Faculdade de Medicina. Departamento e Instituto de Psiquiatria. Núcleo de Epidemiologia Psiquiátrica. São Paulo, SP, Brasil; III Universidade Federal do Espírito Santo. Departamento de Medicina Social. Vitória, ES, Brasil

**Keywords:** Chronic Pain, epidemiology, Mental Disorders, epidemiology, Comorbidity, Health Surveys

## Abstract

**OBJECTIVE:**

To identify the prevalence of 12-month self-reported pain and chronic pain in a general population and to describe their clinical profile to assess if chronic pain is associated with 12-month mental disorders.

**METHODS:**

The data used comes from the São Paulo Megacity Mental Health Survey, a population-based study assessing adult (≥ 18 years) residents of the São Paulo metropolitan area, Brazil. We have assessed the respondents (n = 5,037) using the Composite International Diagnostic Interview (CIDI 3.0), with a global response rate of 81.3%. Descriptive analyses have been performed, and crude and adjusted odds ratios (OR) have been calculated with logistic and multinomial regression and presented with respective 95% confidence intervals (95%CI).

**RESULTS:**

The prevalence of pain and chronic pain in the past 12 months were 52.6% (95%CI 50.3–54.8) and 31.0% (95%CI 29.2–32.7), respectively. Joints (16.5%, 95%CI 15.4–17.5) and back or neck (15.5%, 95%CI 14.2–16.9) were the most frequently reported anatomical sites of chronic pain. On a 10-point analogue scale, the mean intensity of the worst pain was 7.7 (95%CI 7.4–7.8), and the mean average pain was 5.5 (95%CI 5.2–5.6); the mean treatment response was 6.3 (95%CI 6.0–6.6). Mean pain duration was 16.1 (95%CI 15.6–17.0) days a month and 132 (95%CI 126–144) minutes a day. Chronic pain was associated with 12-month DSM-IV mental disorders (OR = 2.7, 95%CI 2.3–3.3), anxiety disorders (OR = 2.1, 95%CI 1.9–3.0), and mood disorders (OR = 3.3, 95%CI 2.4–4.1).

**CONCLUSIONS:**

A high prevalence of chronic pain in multiple sites is observed among the general adult population, and associations between chronic pain and mental disorders are frequent.

## INTRODUCTION

Chronic pain is one of the most frequent reasons for seeking health care^9^, and it is considered a public health problem, causing significant social burden and personal disability^1^ ^,^ ^16^. It is estimated that between 2% and 40% of the adult population suffers from some type of chronic pain around the world^35^. Pain conditions are among the leading causes of years lost due to disability worldwide^40^. Although it is highly prevalent in clinical settings^5^ ^,^ ^16^, epidemiological studies assessing chronic pain in the general population are scarce^12^ ^,^ ^16^ ^,^ ^17^, and they do not usually address cross-cultural differences^9^ or reflect other local realities.

The complex nature of chronic pain (involving multiple psychosocial and genetic factors in its manifestation)^4^, its subjective definition, and the lack of appropriate instruments that assess all types of chronic pain are the main limitations in conducting large-scale population-based surveys^22^. In a review, Harstall and Ospina^12^ have described methodological differences in case definition and case identification related to the minimum duration of chronic pain. While some studies have considered three months, others have used a minimum duration of six months, producing noncomparable prevalence estimates in epidemiological studies. To minimize such variation, the International Association for the Study of Pain recommends, for research purposes, that chronic pain should last for six months or more^18^.

Patients with chronic pain may present symptoms that also occur in depression and anxiety disorders, such as changes in energy, sleep, appetite, and libido, increased irritability, and decreased interest in family, social, and professional activities^16^, imposing difficulties in establishing differential diagnosis. Moreover, chronic pain can be associated with mental disorders – especially depression^7^ ^,^ ^10^, either as cause, consequence, or in a bidirectional manner –, which aggravates the clinical symptoms and turns the clinical management of both conditions more complex^3^. In a study from 19 countries, mental disorders, besides depression, were important predictors of chronic back and neck pain^39^.

The public health importance of chronic pain is incontestable^22^, and epidemiological studies are of great relevance to identify determinants of pain that help increase early identification and control of symptoms, and, therefore, may prevent comorbidity and disability^29^.

This study aims to identify the prevalence of 12-month self-reported pain and chronic pain, to describe the clinical profile of chronic pain, and to assess its association with mental disorders in the adult population living in the São Paulo metropolitan area, Brazil. It contributes to narrow the gap of knowledge regarding the distribution and characteristics of pain and chronic pain in the general population, and it may support prevention policies and control strategies addressing pain and chronic pain in metropolitan Brazil.

## METHODS

This report analyzes data from the São Paulo Megacity Mental Health Survey, a population-based cross-sectional survey of psychiatric morbidity and health-related conditions, assessing a probabilistic sample of residents of the São Paulo metropolitan area, aged 18 years or over. Respondents were selected from a stratified multistage-cluster sampling of households, covering 39 municipalities, without replacement^37^. All respondents were assessed in face-to-face household interviews, after informed written consent was obtained, and total confidentiality was assured. Detailed methodological information has been described elsewhere^37^. The São Paulo Megacity Mental Health Survey was approved by the Ethical and Research Committee of the School of Medicine, Universidade de São Paulo. Data was collected between May 2005 and April 2007, and 5,037 individuals were interviewed, with a global response rate of 81.3%^37^.

Assessment used the World Health Organization (WHO) Composite International Diagnostic Interview (CIDI 3.0)^12^, which is an expanded version of the CIDI 2.1, previously available and validated in Brazil^19^ ^,^ ^27^. The CIDI version 3.0 was developed to be used in the World Mental Health Surveys^15^ and was translated into and adapted to Brazilian Portuguese following international guidelines^36^ ^,^ ^38^. The CIDI 3.0 has clinical and nonclinical sections, producing diagnoses according to the Diagnostic and Statistical Manual of Mental Disorders – Fourth Edition (DSM-IV)^36^.

All sociodemographic information was self-reported and included sex, age at interview (grouped into four age-groups), marital status, occupation (working, student, homemaker, retired, and other), and income, classified into four categories, based on the respondent’s income divided by the median income of the entire sample (low if this ratio was 0.5 or less, low-average if 0.5–1.0, high-average if 1.0–2.0, and high if > 2.0).

The occurrence of any pain in the past 12 months was assessed using questions about physical health, adapted from the U.S. Health Interview Survey^23^. Four questions investigated the occurrence on joint pain, back or neck pain, headache, and pain in other sites. Chronic pain was assessed considering the duration and severity of pain as defined by the criteria of the International Association for the Study of Pain^18^. Although all studies on chronic pain are reliant on self-assessment and self-reporting of symptoms and their intensity, in an attempt to reduce information and recall biases and improve the quality of the data collected, the question to identify the presence of chronic pain was carefully worded: “Serious chronic pain is defined as pain lasting six months or longer that is severe enough either to interfere with your normal activities or to cause emotional distress. With that definition in mind, did you ever have any serious chronic pain in the past 12 months?” If this was endorsed, they were further asked about the pain anatomical sites, as back or neck pain, stomach or abdomen, joint (arms, hands, legs, and feet), face or mandible, chest, headache, and other sites. The prevalence of any chronic pain in the last 12 months was assessed by the identification of chronic pain in at least one anatomical site.

Pain intensity and treatment response were assessed using a 10-point visual analogue scale. For pain intensity, the score zero expressed “no pain” and 10 “the worst pain” in the last 12 months. For treatment response, the score zero expressed “no relief” and 10 “complete relief”. Respondents were encouraged to recall the period over the past 12 months when their pain was more frequent and to give their best estimate on the duration of pain, considering how many days per month and how many hours or minutes per day.

Associations between serious chronic pain and four classes of 12-month DSM-IV mental disorders were assessed. Anxiety disorders included agoraphobia, generalized anxiety disorder, obsessive-compulsive disorder, panic disorder, post-traumatic stress disorder, adult separation anxiety disorder, social phobia, and specific phobia. Mood disorders included major depression, bipolar disorder I and II, and dysthymia. Mental disorders included any anxiety disorder, any mood disorder, any impulse-control disorder (attention deficit/hyperactivity disorder, conduct disorder, oppositional defiant disorder, and intermittent explosive disorder), and any substance use disorder (alcohol abuse or dependence and drug abuse or dependence).

All statistical analyses were performed using Stata, in a survey data framework for complex design datasets (*svy* commands), in which weights were applied to adjust for differences in the probability of selection and nondifferential response, and they were post-stratified according to the age and gender structure of the target population (detailed information on the weighing process is published elsewhere^37^). Crude and adjusted odds ratios (OR) were calculated with logistic regression and presented with respective 95% confidence intervals (95%CI). Differences between proportions of the variables were evaluated using chi-square tests. Multinomial logistic regression models to assess the magnitude of associations were adjusted for demographic variables. Significance level was set at 0.05.

## RESULTS

The sociodemographic characteristics of the total sample and distribution of pain conditions in the previous 12 months are presented in Table 1. The prevalence of any 12-month reported pain was 52.6% (95%CI 50.3–54.8), with higher rates for headache (29.7%, 95%CI 27.7–31.7) and back or neck pain (27.1%, 95%CI 25.9–28.2). Women were over twice more likely to report any pain (OR = 2.2, 95%CI 1.9–2.5). Regarding age, a differential distribution was observed, with lower rates reported by younger respondents, except for headache, which was less frequent among older adults.

**Table 1:** Sociodemographic characteristics of the total sample and of those with self-reported pain in the previous 12 monthsa. (n = 5,037)

Sociodemographic characteristics	Total sample % (95%CI)	Any 12-month pain % (95%CI) n = 2,650	Joint pain % (95%CI) n = 965	Back or neck pain % (95%CI) n = 1,364	Headache % (95%CI) n = 1,495	Other sites % (95%CI) n = 1,172
Total	100%	52.6 (50.3–54.8)	19.2 (18.0–20.5)	27.1 (25.9–28.2)	29.7 (27.7–31.7)	23.3 (22.1–24.4)
Sex		^b^	^b^	^b^	^b^	^b^
Female	53.0 (50.9–55.0)	57.5 (54.5–60.4)	24.4 (22.4–26.3)	33.1 (31.2–35.2)	39.2 (36.3–42.4)	26.4 (24.4–28.3)
Male	47.0 (44.9–49.0)	42.5 (39.5–45.4)	13.5 (12.0–15.0)	20.3 (18.8–21.8)	18.9 (16.6–21.4)	19.8 (17.6–22.1)
Age-groups		^b^	^b^	^b^	^b^	^b^
18–34	44.7 (43.2–46.2)	53.0 (49.8–56.1)	10.5 (8.2–13.2)	21.3 (19.3–23.4)	31.2 (28.1–34.5)	17.6 (16.2–19.2)
35–49	31.8 (30.0–33.6)	43.6 (40.6–75.7)	22.4 (19.9–25.2)	29.0 (25.7–32.5)	33.0 (30.4–35.8)	26.8 (24.0–29.7)
50–64	15.3 (14.2–16.5)	39.0 (34.9–43.2)	32.3 (29.4–35.4)	38.3 (34.2–42.6)	25.8 (22.1–29.7)	30.6 (27.0–34.3)
≥ 65	8.1 (7.1–9.2)	46.7 (38.2–55.4	30.0 (23.9–36.9)	29.7 (22.8–37.7)	15.0 (9.9 -22.2)	26.2 (20.9–32.2)
Marital status		^b^	^b^	^b^		^b^
Married/Cohabitating	58.9 (56.8–61.0)	44.4 (42.2–46.6)	20.5 (18.8–22.3)	28.5 (26.3–30.8)	30.9 (29.1–32.7)	24.9 (23.2–26.7)
Separated/Divorced or Widowed	15.4 (14.3–16.7)	45.6.(41.2–50.0)	27.4 (22.4–33.0)	33.0 (28.7–37.6)	26.5 (22.6–30.9)	26.1 (22.0–30.6)
Never married	25.5 (23.5–27.6)	55.2.(50.5–59.9)	11.3 (9.2–13.8)	20.0 (16.1–24.5)	28.7 (25.1–32.6)	17.5 (14.6–20.9)
Occupation		^b^	^b^	^b^	^b^	^b^
Currently working	63.9 (61.7–65.2)	49.8 (47.4–52.2)	16.8 (15.4–18.3)	25.2 (23.8–26.6)	28.2 (26.0–30.5)	22.2 (21.3–23.2)
Student	1.4 (1.1–1.9)	54.9 (38.3–70.5)	6.4 (2.1–17.9)	6.2 (2.3–15.3)	31.7 (18.4–48.7)	19.7 (10.1–34.8)
Homemaker	13.2 (12.4–14.2)	35.4 (32.2–38.7)	30.1 (25.7–34.8)	37.8 (33.2–42.5)	38.9 (34.0–44.1)	28.7 (25.2–32.4)
Retired	8.1 (7.4–9.0)	45.4 (37.9–53.1)	30.2 (25.0–36.0)	32.3 (27.3–37.8)	17.2 (12.0–24.0)	29.7 (24.0–36.1)
Other	13.5 (12.1–15-1)	47.9 (42.0–53.9)	14.6 (11.2–18.9)	24.2 (20.4–28.4)	34.5 (29.4–40.0)	18.9 (15.1–23.4)
Income		^b^			^b^	
Low income	22.4 (21.2–23.7)	42.8 (38.9–46.9)	21.5 (17.1–26.5)	29.9 (25.8–34.3)	36.7 (32.8–40.8)	24.7 (21.7–28.0)
Low-average income	27.5 (26.4–28.6)	43.7 (39.5–48.1)	19.9 (17.0–23.2)	28.6 (25.5–31.9)	31.9 (27.0–37.3)	23.8 (20.4–27.6)
High-average income	24.3 (22.8–25.9)	49.6 (45.1–54.1)	18.7 (16.5–21.3)	25.1 (21.7–28.9)	25.9 (22.7–29.5)	24.5 (21.9–27.3)
High income	25.6 (24.2–27.1)	53.1 (49.2–57.0)	16.8 (15.0–18.8)	24.6 (22.0–27.4)	24.5 (21.6–27.7)	20.1 (17.2–23.2)

^a^ Weighted data.

^b^ p < 0.01

The prevalence of any chronic pain (Table 2) was 31.0% (95%CI 29.2–32.7, n = 1,561), with higher rates among women compared to men for all anatomical sites, except for pain in the chest. When considering only respondents who had reported any pain in the previous 12 months (n = 2,650), women were more likely to report chronic pain in the face and mandible (OR = 2.6, 95%CI 1.4–4.7) and headache (OR = 2.0, 95%CI 1.4–2.8) compared to men, and men were more likely than women to report chest pain (OR = 0.7, 95%CI 0.6–0.8). The most reported anatomical sites of chronic pain were the joints and the back or neck (Table 2).

**Table 2:** Distribution of self-reported chronic pain in the previous 12 months by anatomical site in the general adult population living in São Paulo, Brazila.

Anatomical site^d^	Among total sample (n = 5,037)				Among those reporting any pain (n = 2,650)			
	
	Total	Female	Male	F/M	Total	Female	Male	F/M
							
	% (95%CI)	% (95%CI)	% (95%CI)	OR (95%CI)	% (95%CI)	% (95%CI)	% (95%CI)	OR (95%CI)
Back or neck pain	15.5 (14.2–16.9)	19.0 (16.8–21.4)	11.6 (10.3–12.9)	1.8 (1.5–2.1)^c^	29.5 (26.7–32.4)	30.9 (27.1–34.9)	27.3 (24.2–30.5)	1.2 (0.9–1.5)
Stomach or abdomen	7.2 (6.4–8.1)	8.6 (7.3–10.0)	5.6 (4.1–7.5)	1.6 (1.1–2.3)^b^	13.7 (12.2–15.3)	14.0 (11.9–16.2)	13.3 (10.2–17.0)	1.1 (0.7–1.5)
Joint pain	16.5 (15.4–17.5)	20.6 (18.9–22.2)	11.8 (10.5–13.2)	1.9 (1.6–2.3)^c^	31.3 (28.8–33.8)	33.4 (30.2–36.7)	27.8 (24.5–31.4)	1.3 (1.0–1.6)
Face or mandible	3.0 (2.4–3.6)	4.5 (3.4–5.7)	1.2 (0.7–1.9)	3.7 (2.0–6.7)^c^	5.6 (4.4–7.0)	7.3 (5.4–9.5)	2.9 (1.8–4.5)	2.6 (1.4–4.7)^c^
Chest	6.3 (5.5–7.0)	6.5 (5.6–7.3)	6.0 (5.1–7.1)	1.1 (0.9–1.3)	11.9 (10.5–13.4)	10.5 (9.2–11.9)	14.2 (11.9–6.8)	0.7 (0.6–0.8)^c^
Headache	9.9 (9.1–10.6)	13.8 (12.1–15.6)	5.4 (4.3–6.7)	2.8 (2.0–3.9)^c^	18.8 (17.3–20.3)	22.5 (19.7–25.4)	12.8 (10.2–15.8)	2.0 (1.4–2.8)^c^
Other	3.4 (2.8–4.0)	4.1 (3.1–5.3)	2.5 (1.8–3.4)	1.6 (1.0–2.5)^b^	6.4 (5.2–7.7)	6.6 (5.0–8.6)	6.0 (4.5–7.8)	1.1 (0.7–1.7)
Any chronic pain	31.0 (29.2–32.7)	36.6 (34.5–38.7)	24.6 (22.3–27.0)	1.7 (1.5–2.0)^c^	58.9 (56.3–61.4)	59.5 (56.2–62.7)	58.0 (53.4–62.3)	1.1 (0.8–1.3)

^a^ Weighted data.

^b^ p < 0.05

^c^ p < 0.01

^d^ Chronic pain defined as pain lasting for 6 months or longer.

Among those reporting any chronic pain, using a 0–10 analogue visual scale, the most intense pain was rated at 7.7 (95%CI 7.4–7.8), the less intense pain was 3.4 (95%CI 3.2–3.5), and average pain was 5.5 (95%CI 5.2–5.6). Regarding treatment response, the mean score of pain relief was 6.3 (95%CI 6.0–6.6). Self-reported pain duration was approximately 16 days a month (95%CI 15.6–17.0) and 132 minutes a day (95%CI 126–144) (Table 3).

**Table 3:** Clinical profile of self-reported chronic pain in the previous 12 monthsa. (n = 1,561)

Chronic pain intensity^b^			Treatment response^b^	Pain duration^b^	

Most intense pain Mean (95%CI)	Less intense pain Mean (95%CI)	Average pain Mean (95%CI)	Pain relief Mean (95%CI)	Days a month Mean (95%CI)	Minutes a day Mean (95%CI)
7.7 (7.4–7.8)	3.4 (3.2–3.5)	5.5 (5.2–5.6)	6.3 (6.0–6.6)	16.1 (15.6–17.0)	132 (126–144)

^a^ Weighted data.

^b^ Assessed using 10-point visual analogue scale. Chronic pain defined as pain lasting for 6 months or longer.

Table 4 shows the sociodemographic correlates of self-reported pain (n = 5,037) and serious chronic pain (n = 2,650). As already seen in Table 1, there were sex and age differences in reporting pain, and age differences in reporting serious chronic pain. Women were twice more likely to report pain compared to men, even after adjusting for age. Older respondents (35–64 years) were more likely to report pain and chronic pain compared to the younger ones (18–34 years) (Table 4). After adjusting for sex, age, occupation, and income, respondents who were separated, widowed, or divorced were less likely to report pain (OR = 0.7, 95%CI 0.6–0.9). Homemakers and respondents with low or low-average incomes were more likely to report pain, but these associations were not significant after adjusting for other sociodemographic characteristics. Apart from age, no other sociodemographic correlates of chronic pain were identified.

**Table 4:** Sociodemographic correlates of self-reported pain and chronic pain in the previous 12 monthsa.

Sociodemographic characteristics	Any 12-month pain^b^ (n = 5,037)				Any 12-month chronic pain^c^ (n = 2,650)			

	OR	95%CI	OR adjust	95%CI	OR	95%CI	OR adjust	95%CI
Sex^d^								
Male	1		1		1		1	
Female	2.2	1.9–2.5^j^	2.2	1.9–2.5^j^	1.1	0.8–1.3	1.1	0.8–1.4
Age group^e^								
18–34	1		1		1		1	
35–49	1.4	1.3–1.7^j^	1.4	1.2–1.6^j^	1.5	1.2–1.9^j^	1.5	1.3–1.9^j^
50–64	1.8	1.4–2.2^j^	1.7	1.4–2.2^j^	1.5	1.2–2.0^j^	1.4	1.0–1.8^i^
≥ 65	1.3	0.9–1.9	1.3	0.8–2.0	1.2	0.7–1.8	0.8	0.5–1.2
Marital status^f^								
Married/Cohabitating	1		1		1		1	
Separated/Widowed/Divorced	0.9	0.8–1.1	0.7	0.6–0.9^j^	1.1	0.8–1.4	1.1	0.8–1.4
Never married	0.6	0.5–0.8^j^	0.8	0.6–1.0	0.9	0.7–1.1	0.9	0.7–1.2
Occupation^g^								
Working	1		1		1		1	
Student	0.8	0.4–1.5	1.0	0.5–1.9	1.0	0.4–2.7	1.1	0.4–2.9
Homemaker	1.8	1.5–2.1^j^	0.9	0.7–1.2	1.2	0.9–1.6	1.1	0.8–1.5
Retired	1.2	0.9–1.6	1.0	0.8–1.3	1.5	1.0–2.1^i^	1.3	0.8–2.0
Other	1.1	0.8–1.4	1.0	0.7–1.4	0.8	0.6–1.0	0.8	0.6–1.0
Income^h^								
Low income	1		1		1		1	
Low-average income	1.0	0.7–1.2	1.0	0.8–1.3	0.8	0.6–1.0	0.8	0.6–1.0
High-average income	0.8	0.6–0.9^i^	0.8	0.6–1.0	1.0	0.7–1.3	1.0	0.7–1.3
High income	0.7	0.5–0.8^j^	0.7	0.7–0.9	0.9	0.7–1.2	0.9	0.7–1.1

^a^ Weighted data.

^b^ Any 12-month pain in the total sample.

^c^ Any 12-month chronic pain among those with any 12-month pain.

^d^ Adjusted by age.

^e^ Adjusted by sex, marital status, occupation, and income.

^f^ Adjusted by sex, age, occupation, and income.

^g^ Adjusted by sex, age, marital status, and income.

^h^ Adjusted by sex, age, marital status, and occupation.

^i^ p < 0.05

^j^ p < 0.01

Respondents with chronic pain in all anatomical sites were more likely to present a 12-month DSM-IV mental disorder (any anxiety, any mood, and any mental disorder), with OR ranging from 2.1 to 5.6 (Table 5). After adjusting for age, sex, income, occupation, and marital status, those with any chronic pain were 2.3 times (95%CI 1.9–3.0) more likely to have a 12-month DSM-IV anxiety disorder, 3.3 times (95%CI 2.6–4.4) more likely to have a 12-month DSM-IV mood disorder, and 2.7 times (95%CI 2.3–3.3) more likely to have a 12-month DSM-IV mental disorder (Table 5). For all classes of mental disorders, chronic pain in the joints, in the back or neck region, and headache were the most frequently reported anatomical sites.

**Table 5:** Prevalence of chronic pain by anatomical site and associations with DSM-IV mental disorders in the previous 12 monthsa.

Anatomical site	Any anxiety disorder - chronic pain			Any mood disorder - chronic pain			Any mental disorder - chronic pain		
		
	% (95%CI)	Crude OR (95%CI)	Adj. OR (95%CI)^b^	% (95%CI)	Crude OR (95%CI)	Adj. OR (95%CI)^b^	% (95%CI)	Crude OR (95%CI)	Adj. OR (95%CI)^b^
Back or neck pain	26.1 (22.8–29.6)	2.3 (2.0–2.8)^c^	2.1 (1.8–2.6)^c^	33.4 (28.9–39.1)	3.3 (2.5–4.3)^c^	3.2 (2.4–4.1)^c^	26.0 (22.6–29.6)	2.7 (2.1–3.4)^c^	2.7 (2.1–3.3)^c^
Stomach or abdomen	13.0 (10.1–15.8)	2.3 (1.6–3.3)^c^	2.2 (1.5–3.1)^c^	19.2 (15.0–23.9)	3.2 (2.7–5.3)^c^	3.7 (2.7–5.0)^c^	12.9 (10.4–15.8)	2.8 (1.9–4.0)^c^	2.7 (1.9–3.8)^c^
Joint pain	29.0 (25.1–33.1)	2.6 (2.1–3.1)^c^	2.4 (1.9–2.9)^c^	34.0 (27.7–40.6)	3.0 (2.2–4.1)^c^	2.9 (2.1–4.0)^c^	27.6 (24.1–31.3)	2.7 (2.2–3.9)^c^	2.7 (2.2–3.4)^c^
Face or mandible	6.3 (4.3–9.0)	2.9 (1.9–4.4)^c^	2.5 (1.6–3.9)^c^	11.0 (7.9–13.9)	5.6 (3.6–8.0)^c^	5.0 (3.1–8.1)^c^	7.0 (5.2–9.3)	5.3 (3.4–8.2)^c^	5.1 (3.2–8.2)^c^
Chest	12.0 (9.8–14.4)	2.6 (1.9–3.5)^c^	2.6 (1.9–3.5)^c^	14.9 (11.0–19.7)	3.1 (2.1–4.6)^c^	3.3 (2.2–4.9)^c^	12.1 (10.1–14.3)	3.3 (2.4–4.5)^c^	3.4 (2.5–4.7)^c^
Headache	18.0 (14.3–22.8)	2.6 (1.7–3.9)^c^	2.2 (1.4–3.3)^c^	27.3 (23.5–31.3)	4.4 (3.4–5.7)^c^	3.7 (2.9–4.8)^c^	18.5 (16.0–21.3)	3.2 (2.4–4.3)^c^	2.9 (2.2–3.8)^c^
Other	7.0 (5.1–9.4)	2.9 (1.8–4.5)^c^	2.8 (1.7–4.6)^c^	7.6 (5.1–11.0)	2.8 (1.7–4.5)^c^	3.0 (1.8–5.0)^c^	6.3 (4.6–8.5)	2.9 (1.7–5.0)^c^	3.2 (1.9–5.4)^c^
Any chronic pain	52.1 (48.8–55.4)	2.4 (2.0–2.9)^c^	2.3 (1.9–3.0)^c^	57.7 (51.6–63.5)	3.5 (2.6–4.7)^c^	3.3 (2.6–4.4)^c^	47.6 (44.7–50.4)	2.7 (2.3–3.3)^c^	2.7 (2.3–3.3)^c^

^a^ Weighted data.

^b^ OR: Odds ratio adjusted by sex, age, marital status, occupation, and income.

^c^ p < 0.01

## DISCUSSION

One-third of the general adult population reported important chronic pain in the previous 12 months (31%). This finding is similar to reports from other countries participating in the WMH surveys (37.7% in developing and 38.9% in developed countries)^35^, and among primary care patients in the city of Rio de Janeiro (30.8%)^9^, Brazil. Higher rates were reported in the population of Salvador, State of Bahia (41.4%)^29^, Brazil, among public servants of the Universidade Estadual de Londrina, State of Paraná (61,4%)^16^, Brazil, and in older people living in the catchment area of a primary care unit in Londrina, State of Paraná, Brazil (62.2%)^5^. Very low and discrepant rates were reported among patients (2.7%) attending a primary health care unit in Guarulhos, State of São Paulo^21^, Brazil.

Differences in prevalence estimates of chronic pain can be partly explained by the different research methods applied, such as the assessment and definition of chronic pain and the selection procedures. Furthermore, cultural differences on coping strategies can influence prevalence rates, as suggested by Gureje et al.^9^ The sociodemographic structure of the populations studied can also influence prevalence rates. Miranda et al.^20^ have suggested that older adults are more likely to suffer from chronic pain, as a result of the aging process and physical ilnesses^29^, while the presence of pain in middle-aged persons is more likely to be reported, as this is usually an economically active population and pain may be triggered by occupational activities or cause working impairment^29^.

The mean intensity of average chronic pain found in this study (5.5; SE = 0.07) was lower (7.4; SE = 2.16) than that reported by Ruviaro and Filippin^28^. Overall, reports on the clinical profile of chronic pain in the general population are very scarce, limiting comparisons. The most prevalent anatomical sites of self-reported pain are the joints and back and neck, and this is consistent with other studies^9^ ^,^ ^26^ ^,^ ^29^. Chronic back pain is the leading cause of years lost due to disability worldwide^40^, and it is also the leading cause of occupational disability for those aged up to 45 years in developed countries, imposing high costs related to the use of health care facilities and treatment^7^.

Regarding sex, women had higher rates of self-reported pain, which is commonly reported in the literature^5^ ^,^ ^9^ ^,^ ^16^ ^,^ ^29^. However, the relationship between sex and the occurrence of pain differs from one condition to another, and it varies across the life cycle. Although, for most anatomical sites, women are more likely to report pain compared to men, this is not the case for all pain conditions at every stage of life^17^. Higher rates in women may be due to a higher biological sensitivity to stimuli, as women might detect pain signals that men do not notice^17^. At a cognitive level, the threshold for labeling stimuli as painful is generally lower for women compared to men^17^. Another important contributing factor may be related to the social and cultural differences in the upbringing of boys and girls, as it is more acceptable for girls and women to report experiencing pain^17^. Furthermore, because of the accumulation of functions performed by women, within the family, at work, and in society, disability may represent a greater threat, resulting in increasing search for medical care^16^.

Regarding the association between chronic pain and mental disorders, our study confirms findings from other researchers, in which respondents with chronic pain are twice more likely to present mental disorders, especially mood disorders^3^ ^,^ ^6^ ^,^ ^9^ ^,^ ^10^. There are variations according to the anatomical site affected, as found in this study, such as a high and significant association between anxiety disorders and chest pain (OR = 2.6, 95%CI 1.9–3.5) or joint pain (OR = 2.4, 95%CI 1.9–2.9). Moreover, there are significant associations between mood disorders and face or mandible pain (OR = 5.0, 95%CI 3.1–8.1), headache (OR = 3.7, 95%CI 2.9–4.8), stomach (OR = 3.7, 95%CI 2.7–5.0), and back or neck pain (OR = 3.2, 95%CI 2.4–4.1).

The main causes of chest pain are diverse, but psychological factors commonly influence chest pain expression in patients with or without heart disease^30^. Patients with panic and other anxiety disorders commonly present chest pain, leading them to constant seek cardiologic consultations, and this may result in later identification of the psychiatric condition^31^. Physicians and other health professionals must be aware of psychological and psychopathological influences on chest pain expression to provide optimal treatment to their patients^30^.

Although often neglected in clinical settings, the association between chronic pain and mood disorders, especially depression, are commonly reported worldwide^3^ ^,^ ^8^ ^,^ ^41^, and it is even greater among patients with pain in multiple sites^9^. Moreover, patients with disabling chronic pain may develop depressive symptoms^25^, as everyday simple activities, such as walking or using stairs, can become difficult achievements, leading to individual dependence and social isolation. In a reverse causal model, different types of pain may be part of the clinical expression of depression^24^.

Chronic pain in the face or temporomandibular joint can also relate to changes in emotional functioning and may impair daily living activities, and depression is the mental disorder most commonly associated with this type of pain^8^. The etiology of temporomandibular disorder is complex and multifactorial, and emotional distress is considered the main causal factor related to its occurrence^33^.

Studies tend to report a global association between chronic pain and emotional disturbance, but there are differences according to each clinical condition and individual emotional susceptibility^41^. These relationships are difficult to disentangle, since pain process involves many mechanisms, such as interactions in the central nervous system neurotransmitters and receptors, genetic influences, and inhibition of pain circuitry, contributing to the complexity of studying and understanding pain processes^4^.

The symptoms of chronic pain may be related to symptoms of mental disorders, suggesting that preexisting features may be activated at the beginning of the pain processes, and the stress associated with chronic pain can trigger the onset of mental disorders in predisposed individuals^6^ ^,^ ^14^. On the other hand, chronic pain can also be an important manifestation of mental disorders^25^. The association between chronic pain and mental disorders can occur simultaneously or can be bidirectional, but, in all cases, the need of multidisciplinary care is frequent^2^ ^,^ ^3^. The cross-sectional design of this study cannot allow the identification of the causal direction of associations, although a survival analysis of cross-sectional studies has reported that most prior mental disorders are associated with later onset of pain^39^.

It is also important to emphasize some limitations, which may influence the interpretation of the findings. The occurrence of chronic pain was self-reported, using a limited number of questions based on a stated definition of “serious chronic pain”, which might have led to the underestimation of mild pain conditions, as more severe and lasting pain is more likely to be reported^13^. However, previous findings have suggested that self-reporting might be less distorted than behavioral pain measures, which are likely to be influenced by cultural and social norms or coping strategies^11^ ^,^ ^32^. Additionally, self-report methods have shown to present moderate to high agreement with medical records^23^. Since pain may be associated with premature death, these findings may have been affected by survival bias. Moreover, the most severe and impairing cases were probably not included in the survey, as they either would not be able to participate in such a complex assessment or may have been admitted into healthcare facilities; in both cases, this would have led to underestimation of the strength of the associations with the mental disorders examined. Retrospective information regarding mental health symptoms, collected in a cross-sectional design, is likely to be susceptible to recall bias, which may result in underestimating diagnosis of psychiatric disorders^4^ ^2^. In order to minimize this bias, only mental disorders and pain conditions occurring over the previous 12-month period were included. Finally, the investigation of such associations did not consider the temporal sequence of events, leaving it unclear whether mental disorders precede or follow the development of chronic pain, or both.

In conclusion, a high prevalence of chronic pain in multiple sites is observed among the general adult population, and associations between chronic pain and mental disorders are frequent. Therefore, it is important to carefully evaluate patients with mood and anxiety disorders, screening for pain conditions, and vice versa, to provide an accurate diagnosis and to ensure adequate treatment. Improving the knowledge on correlates, clinical profile, and comorbidities of chronic pain in the general population can better guide prophylactic strategies and early identification and, thus, reduce the global burden associated with chronic pain conditions, in this way decreasing individual suffering and societal costs. The results of this study reinforce the need for interaction between different areas of clinical expertise to ensure the best pain management to those affected, taking into account the biological, emotional, cultural, and social aspects involved in pain conditions.
